# The Charge of Lay Control Demonstrably a Myth

**Published:** 1917-02-03

**Authors:** 


### MORE ABOUT THE ROYAL BRITISH COLLEGE OF NURSING.

### The Charge of Lay Control Demonstrably a Myth.

When we concluded our article last week on the Royal British College of Nursing by directing attention to the "pedantry or worse" which objected to the inclusion of the "lay" element on its Council, we ventured to say that "no doubt more would be heard" of the statement of the Chairman of the Executive Committee of the Society for the State Registration of Nurses that "the professional status of the R.B.N.A. would, under the Supplemental Charter, be absorbed into a Lay Corporation, as it provided for the inclusion of the laity as members of the College without any qualification"; and the expected has happened. In her editorial on Saturday last Mrs. Bedford Fenwick returns to the topic, and alleges that, if the R.B.N.A. and the College of Nursing are amalgamated, "British Nurses will no longer possess a Professional Association organised under a Royal Charter," but will become members of the Royal British College of Nursing, "an educational body, the constitution of which admits the laity to membership and official control." Perhaps, therefore, it may be worth while to probe this matter a little more deeply.

We have already urged that only at the expense of some clarity of definition can doctors be described as "Professional" members of a Society of *Nurses*, and Mrs. Bedford Fenwick herself lends support to our contention by remarking in a footnote to the report of the R.B.N.A. meeting given in her Journal of the 27th ultimo, that "She founded the Association in 1887 as a British *Nurses'* Association, and has always disapproved of a monopoly of power by the honorary Medical Officers."

Without pausing to consider the literal accuracy of either of these statements, we pass on by dry facts and figures to point out that on the Council of the Royal British College of Nursing as set out in the Supplementary Charter there are thirty-nine members, of whom two only (the Hon. Arthur Stanley and another) are "laymen." Moreover, as Mr. Stanley said at Leeds, the original Council remains in office for a bare two years, at the end of which time every member of the College has a vote in the election of the new Council. What, then, becomes of the nonsense—for it is nothing else—about the official control of the laity? If they are not wanted, the laymen can be promptly voted off the Council, and they certainly can look for little help from the other laymen who are members of the Royal British College of Nursing. The R.B.N.A. has none whom Mrs. Bedford Fenwick reckons as "laymen" on its Roll, and the College of Nursing has just the seven laymen who, with the exception of Mr. Stanley, disappeared from the scene when they had appointed the first fifteen members of the Council, and in addition to them the six "lay" persons—all, we may say parenthetically, Governors of important nurse-training schools—who sit on the Scottish Board. In all, even if the Irish Board should have as many laymen on it as the Scottish Board, there will be fewer than twenty-five laymen, all told, out of a membership of the R.B.C.N., which must already run into more than 5,000 nurses.

Now let us look at the "lay" question from another point of view. The College is a stepping-stone, we are all agreed, to Legal Registration. If the Council, fully elective in character, ought not to admit even such "laymen" as it desires to have "in the interests of the College," *a fortiori* the General Nursing Council, which is a legal body, keeping a legally protected Register, and with far stronger coercive powers than any that a voluntary Association can have over its members, must have none but nurses on its Governing Body, if nurses are to be saved from the arrogant pretensions and "control of the laity." Clearly here, if anywhere—accepting Mrs. Bedford Fenwick's point of view—we must avoid at all costs such a constitution of the "General Council for the Training and Education of Nurses" as would "admit the laity to membership and official control." Alas! brought up against the hard wall of "things as they are," the Central Committee for the State Registration of Nurses, in the latest edition of their Registration Bill which, as we are assured, holds the field, concedes on the permanent Council of thirty-three persons no fewer than seven places which may be filled by the "laity"—three appointed by the Privy Council, and four by the Nurse-Training Schools attached to hospitals approved by the Council. Out of thirty-three Councillors there are but sixteen direct Representatives of the Women Nurses on the General Register, and, after all that has been said to their disparagement, it is a little astonishing to find that the nurses are bound to elect as eight of these Representatives either past or present *Matrons* of recognised Nurse-Training Schools. Could this humbug of inconsistency be more convincingly revealed?

### New Bye-Laws of R.B.C.N.

It is not, however, our object to-day to submit the Central Committee's Bill to further destructive analysis. That may come later. Just now we wish to proceed with our review of the new bye-laws of the Royal British College of Nursing, many of which are so much in the ordinary course of things as not to call for comment. The third bye-law defines the conditions of membership, limiting it to nurses on the Register, members of Council, or Local Boards, and persons elected by the Council in "the interests of the Corporation." We have already amply justified the admission of the last-named class, but it is of some importance perhaps to note that by Bye-law 27 (*b*) persons who are members of the Corporation by reason only of being members of the Council or Local Boards, *ipso facto* cease to be members of the Corporation when they cease to be members of the Council or Local Board.

The conditions under which the Council may remove unworthy members are formulated under Bye-law 26, which, expanding Clause 60 (9) of the Articles of Association of the College, requires fourteen days' notice of the proceedings to be served on the person inculpated, and gives her a right to be heard in her defence in person or by agent. Having regard to the grievance which has been manufactured out of the bald statement of the College Article that nurses may be removed from the Register " at the discretion of the Council," it was, we think, wise to set out the safeguards that, in any case the Council would have had to take, were it not prepared to see its decision to remove a nurse set aside by a Court of Law. It may, however, be remarked that the College requires a two-thirds majority of the *whole Council* (twenty-seven) —a fact to which attention was carefully *not* called, and the removal bye-law, a two-thirds majority of members *present and voting*, the quorum being twelve.

Bye-law 8 is directed against a frivolous abuse by members of the privilege of summoning Special General Meetings. It lays down that the demand to the Chairman of the Council to summon a meeting shall be signed by at least a hundred members of the College and a quarter of the members of the Council. We understand that there were times in the history of the R.B.N.A. when such a safeguard would have been very valuable to prevent waste of time and money.

Under Bye-law 10 the Provisional Council has to formulate a scheme for the future constitution and election of the Permanent Council, at latest within eighteen months of coming into existence, and before coming into force the scheme must receive the approval of the Privy Council. Bye-laws 16 and 17 provide for a Consultative and Examination Board on the lines of the College Articles of Association.

Finally, Bye-law 20 prescribes that the Council shall keep a Register of Nurses, and that the first Register shall be formed by the fusion of the existing Registers of the R.B.N.A. and the College of Nursing, Limited. Thus we are brought back to the point from which we set out—namely, the duty of every qualified nurse to register, and to induce her friends to register. Already the response to our appeal has been wonderful, beyond all expectation. Wars are won by munitions and men. We may trust Mr. Stanley and his friends for the munitions. It is for the nurses themselves to man the fighting-line, and to provide reserves—and more reserves.

